# Supplementary material for: Global hypo-methylation in a proportion of glioblastoma enriched for an astrocytic signature is associated with increased invasion and altered immune landscape
Source: eLife. 2022 Nov 22;11:e77335. doi: 10.7554/eLife.77335 (PMC9681209; doi:10.7554/eLife.77335)
Supplement: Figure 2—source data 1. [file elife-77335-fig2-data1.zip › Figure_2_source_data_1/Figure_2I_J/homerResults/motif32.similar.html]

motif32

## Information for motif32

A
C
G
T
A
G
T
C
T
C
A
G
A
G
T
C
G
C
A
T
T
C
G
A
G
T
C
A
G
T
A
C
  
Reverse Opposite:  

C
A
T
G
C
A
G
T
A
G
C
T
C
G
T
A
T
C
A
G
A
G
T
C
T
C
A
G
G
T
C
A
  

|  |  |
| --- | --- |
| p-value: | 1e-29 |
| log p-value: | -6.766e+01 |
| Information Content per bp: | 1.632 |
| Number of Target Sequences with motif | 37.0 |
| Percentage of Target Sequences with motif | 5.10% |
| Number of Background Sequences with motif | 1.5 |
| Percentage of Background Sequences with motif | 0.53% |
| Average Position of motif in Targets | 89.2 +/- 56.9bp |
| Average Position of motif in Background | 136.0 +/- 4.2bp |
| Strand Bias (log2 ratio + to - strand density) | 0.0 |
| Multiplicity (# of sites on avg that occur together) | 1.06 |
| Motif File: | file (matrix) reverse opposite |

### Similar de novo motifs found

|  |  |  |  |  |  |  |  |
| --- | --- | --- | --- | --- | --- | --- | --- |
| Rank | Match Score | Redundant Motif | P-value | log P-value | % of Targets | % of Background | Motif file |
| 1 | 0.602 | A G C T C G T A A C G T A G T C A C T G C G T A | 1e-19 | -44.329433 | 3.86% | 0.65% | motif file (matrix) |
| 2 | 0.785 | A C T G A C G T A C G T C G T A C T A G A G T C | 1e-13 | -30.553481 | 5.79% | 1.76% | motif file (matrix) |
| 3 | 0.712 | A G T C A C G T A G T C A C T G A G T C A C G T | 1e-8 | -19.711058 | 3.99% | 1.32% | motif file (matrix) |
| 4 | 0.646 | A G T C A C G T A G T C A C G T C G T A C G T A | 1e-6 | -15.880519 | 8.68% | 4.59% | motif file (matrix) |
